# The Effects of Dithiothreitol on DNA

**DOI:** 10.3390/s17061201

**Published:** 2017-05-24

**Authors:** Søren Fjelstrup, Marie Bech Andersen, Jonas Thomsen, Jing Wang, Magnus Stougaard, Finn Skou Pedersen, Yi-Ping Ho, Marianne Smedegaard Hede, Birgitta Ruth Knudsen

**Affiliations:** 1 Department of Molecular Biology and Genetics, Aarhus University, 8000 Aarhus, Denmark; soeren.fjelstrup@inano.au.dk (S.F.); marieba89@gmail.com (M.B.A.); jo07@mbg.au.dk (J.T.); jwang@mb.au.dk (J.W.); fsp@mbg.au.dk (F.S.P.); ypho@ee.cuhk.edu.hk (Y.-P.H.); 2 Interdisciplinary Nanoscience Center (iNANO), Aarhus University, 8000 Aarhus, Denmark; 3 Department of Pathology, Aarhus University Hospital, 8000 Aarhus, Denmark; magnstou@rm.dk; 4 Division of Biomedical Engineering, Department of Electronic Engineering, The Chinese University of Hong Kong, Shatin, NT, Hong Kong, China; 5 Zymonostics ApS, 8000 Aarhus, Denmark

**Keywords:** single molecule detection, DTT, DNA modifying enzyme, DNA sensor, thiol, DNA nicking

## Abstract

With the novel possibilities for detecting molecules of interest with extreme sensitivity also comes the risk of encountering hitherto negligible sources of error. In life science, such sources of error might be the broad variety of additives such as dithiothreitol (DTT) used to preserve enzyme stability during in vitro reactions. Using two different assays that can sense strand interruptions in double stranded DNA, we here show that DTT is able to introduce nicks in the DNA backbone. DTT was furthermore shown to facilitate the immobilization of fluorescent DNA on an NHS-ester functionalized glass surface. Such reactions may in particular impact the readout from single molecule detection studies and other ultrasensitive assays. This was highlighted by the finding that DTT markedly decreased the signal to noise ratio in a DNA sensor based assay with single molecule resolution.

## 1. Introduction

In recent years, game changing technical advancements within the field of biosensors have enabled researchers to measure the activity of DNA modifying enzymes with ultra-high sensitivity and to detect even a single DNA modification event [1,2]. With the emergence of these tools for studying rare events, the importance of unexpected and infrequent side reactions mediated by additives such as dithiothreitol (DTT) and not the main reactants, such as the DNA modifying enzyme itself, becomes increasingly important.

DTT is a potent reducing agent widely exploited in molecular biology as an enzyme stabilizing agent and can be found in the supplied reaction buffers of many commercially available DNA modifying enzymes as well as in their storage buffers (see Supplementary Materials Table S1). The main role of DTT in molecular biological assays is to keep proteins in a reduced state [3,4]. Thiol containing compounds have, however, also been shown to be very effective at protecting DNA from irradiative damage [5,6,7,8], which is thought to be due to their ability to scavenge oxygen and nitrogen radicals. Ironically, in addition to its role as a DNA protective radical scavenger, DTT is also a potent inducer of DNA damage since, at certain concentrations, thiols in general have the ability to produce oxidative species, such as the hydroxyl radical [9,10,11], which has been shown to induce DNA breaks as well as other kinds of damage on DNA molecules [12]. In agreement with this finding, thiols have been linked to chromosome damage and apoptosis in cells [13,14]. Thiol induced generation of hydroxyl radicals is believed to be due to thiols boosting a Cu^2+^ catalyzed mechanism analogous to the oxygen radical generating reaction known as the Haber–Weiss reaction. By the proposed mechanism, Cu^2+^ catalyzes a reaction where thiols are oxidized by molecular oxygen which, as a result, is reduced to O_2_^−^. By reaction with H^+^, O_2_^−^ is subsequently converted into the highly reactive hydroxyl radical in a reaction including an H_2_O_2_ intermediate [11,15,16]. Cu^2+^/thiol induced DNA damage has been shown for both monothiols and dithiols but, in the present study, the focus is on the dithiol DTT due to its extensive use in DNA based studies (see Supplementary Materials Table S1).

Motivated by the emergence of single molecule detection methods and the presence of DTT in virtually all traditionally used DNA modification protocols, we set off to elucidate the effect of DTT on DNA and thereby its influence on the results of highly sensitive DNA based assays. Examples of such assays include polymerase-based amplifying protocols such as PCR and the Rolling Circle Amplification (RCA) method, which has been developed into a single molecule detection scheme by combining it with fluorescence labelling. Surprisingly, we found that DTT is able to introduce single stranded nicks in covalently closed double stranded DNA circles even without any addition of catalyst. These DTT generated nicks were shown to be able to function as unintended starting points for RCA which forms the basis of many modern ultrasensitive assays [17,18,19,20,21]. Furthermore, DTT was able to immobilize DNA to NHS-ester coated microscopy slides used for single molecule studies of DNA modifications. The consequence of these unexpected side-effects of DTT was highlighted by its ability to increase the background in a single molecule detection assay using a DNA based sensor system developed to measure retroviral integrase (IN) activity.

## 2. Materials and Methods

### 2.1. DNA-Oligonucleotides

Primer 1: 5′-ATTTTTCTAAGTCTTTTAGATCGAACGACTCAGAATGATGCATGTATACTAAACTCACAAATTAGAGC-3′
Primer 2: 5′-TTTTTTTTTTTTTTTTTTTTTTTTTGCTTTCTCATAGCTCACGCTG-3′
IN-fw: 5′-AACTGGCGCGCCATGGCTTCTGAC-3′
IN-rv: 5′-TTAATCTTCGTCCTGACGAGAAGCAACG-3′
5′-Amine-Oligo A: 5′-Am-TTTAGTCAGTGTGGAAAACTCTAGCAGT-3′
Oligo B: 5′-ACTGCTAGAGATTTTCCACACTGACTAAA-3′
Acceptor-circle-fw: 5′-CCGCCCTGCAGCCTCAATGCACATGTTTGGCTCCC-3′
Acceptor-circle-rv: 5′-TAATTCTGCAGACGATAGCGGTACATCTCGG-3′
Detection probe: 5′-FAM-CCTCAATGCACATGTTTGGCTCC-3′

All oligonucleotides were purchased from Sigma-Aldrich (St. Louis, MO, USA).

### 2.2. Nicking of Supercoiled Plasmid

200 fmol of supercoiled pBR322-plasmid was incubated with 0, 0.1, 1, or 10 mM DTT at 37 °C for 20 h in a reaction buffer containing 10 mM Tris-HCl pH 7.5, 300 mM NaCl, and 1 mM EDTA. The reaction products were separated in a 1% agarose gel (containing 0.5 µg/mL ethidium bromide) run at 100 V for 2 h. The bands were visualized using the Bio-Rad Universal Hood II Gel Doc System and the intensity of the individual bands was quantified using ImageJ.

### 2.3. Polymerase Enabled Nick Detection (Modified Nick Translation Assay)

50 ng of the plasmid pDsRed-monomer-CI (BD Biosciences) was incubated at 37 °C for 20 h with 0, 0.1, 1, or 10 mM of DTT in Tris buffered saline (10 mM Tris-HCl pH 7.5, 300 mM NaCl, and 1 mM EDTA) in a 10 µL reaction volume. After reaction with DTT, the DNA was purified using the E.Z.N.A.^®^Cycle Pure Kit. The purified DNA was incubated with 1U of DreamTaq polymerase (Thermo Scientific), 200 µM of each dNTP (of which, 2 nM of the dATP was [alpha-32P] from Perkin Elmer) in the supplied polymerase reaction buffer. The reaction mixture was incubated at 72 °C for 1 h after which the reaction was stopped by addition of proteinase K to a final concentration of 1 µg/µL and incubated for 30 min at 37 °C. The DNA was ethanol precipitated and dissolved in 30 µL of TE buffer (10 mM Tris pH 7.5, 1 mM EDTA). The samples were diluted with 70 µL ddH_2_O and heated to 95 °C for 10 min. After cooling on ice, 100 µL of 1 M NaOH was added. The solution was then incubated at room temperature for 20 min. Using a dot blot apparatus, the samples were transferred to a piece of Hybond-XL membrane (Amersham) which had been pre-wetted with 2× SSC (300 mM NaCl in 30 mM sodium citrate pH 7.0) for 5 min. The membrane was washed in 2× SSC, removed from the apparatus, and finally washed in 2× SSC at room temperature for 30 min. It was then allowed to air dry and placed on a Phosphorimager screen which was exposed for 2 h and then scanned using a SF Molecular Dynamics Phosphorimager.

### 2.4. DNA Adhesion Assay

For the DNA adhesion assay, a fluorescently labelled 1500 bp PCR product was produced using DreamTaq polymerase and DreamTaq buffer (ThernoFisher, Waltham, MA, USA) supplemented with 200 µM of each dNTP. pYES2.1 (ThernoFisher, Waltham, MA, USA) was used as the template, and the primers 1 and 2 (see the DNA-oligonucleotides section) as forward and reverse primers, respectively. The reaction was spiked with 20 μM Aminoallyl-dUTP-XX-ATTO-488 (Jena Bioscience, Jena, Germany). This should result in 30–40 fluorophores being incorporated into the PCR product. After 30 PCR cycles, the PCR product was purified using the E.Z.N.A.^®^ Gel Extraction kit (Omega Biotek, Norcross, GA, USA).

The PCR product was diluted to 0.25 nM in a buffered saline solution (10 mM Tris-HCl pH 7.5, 300 mM NaCl, and 1 mM EDTA) with or without 10 mM DTT. The DNA solutions were allowed to incubate for 1 h at 37 °C on an NHS-activated glass slide (CodeLink^®^ Activated slides, Surmodics, Eden Prairie, MN, USA), blocked as directed by the manufacturer. The slide was subsequently washed in wash buffer A (0.1 M Tris-HCl pH 7.5, 150 mM NaCl, and 0.3% SDS) for 20 min, wash buffer B (0.1 M Tris-HCl pH 7.5, 150 mM NaCl, and 0.05% Tween-20) for 10 min, 10 mM Tris-HCl pH 8.8 for 5 min, and finally dehydrated with 96% ethanol for 1 min. The slide was air-dried and mounted using Vectashield (Vector Laboratories, Burlingame, CA, USA). Immobilized DNA molecules were visualized using fluorescence microscopy (Olympus IX73—Olympus Corporation, Tokyo, Japan). Light source: X-Cite 120 Q (120 W mercury vapor short arc); Camera: Andor Zyla; Filtercube: U-FBNA (excitation filter: 470–495, emission filter: 510–550); Objective: UPLSAPO 60XO (NA = 1.35); Exposure time: 300 ms (all purchaged via Olympus Corporation, Tokyo, Japan).

For each experimental setup, nine images were taken and the number of fluorescent signals per microscopic image (277 × 234 µm^2^) was determined using ImageJ. The images were analyzed blindly by adjusting the threshold to fit the signals observed and then using the “analyze particles” function (size: 20–200 pixel^2) to count the number of signals. The average number of signals per image frame was finally calculated.

### 2.5. Purification of IN

The HIV integrase gene was cloned and the protein expressed and purified as described in [22]. A plasmid for expression of IN was made by PCR amplification of the IN gene from pEGFP-PK-IN (Addgene: pPS2986, Cambridge, MA, USA) using primers IN-fw and IN-rv, (see the DNA oligonucleotide section above). The PCR product was cloned into the expression vector pTrcHis-TOPO using the pTrcHis TOPO^®^ TA Expression Kit (Invitrogen, Carlsbad, CA, USA). The resulting plasmid, pTrcHis-TOPO-HIV_IN, was amplified in BL21 E. coli cells and expression of IN was induced at OD 0.6 using 0.3 mM IPTG. After 3 h incubation at 30 °C, the cells were pelleted and resuspended in ice-cold solubilization buffer (50 mM sodium phosphate buffer, pH 8.0, 300 mM NaCl, 10 mM imidazole, 10 mM Chaps (3-[(3-Cholamidopropyl) dimethylammonio]-1-propanesulfonate), and one protease inhibitor EDTA-free tablet (Roche) per 50 mL of buffer). The cells were lysed by addition of 2.5 mg/mL lysozyme, incubation on ice for 15 min, and 5 × 15 s sonication until the lysate appeared clear. To remove cell debris, the lysates were centrifuged at 15,000 g for 1 h at 4 °C and the supernatant (containing the IN) was transferred to a clean centrifuge tube.

IN was purified by fast protein liquid chromatography (FPLC) using columns packed with 3 mL of the Ni-NTA Superflow (Qiagen) Ni^2+^ resin. Prior to loading the protein samples, the column was washed with 10 column volumes of ddH_2_O and equilibrated with 10 column volumes of equilibration buffer (10 mM Tris-HCl, pH 7.5, 200 mM NaCl, 5 mM MgCl_2_, 10% glycerol, 1 mM PMSF) (flow rate: 0.5 mL/min). The lysate was diluted 1:1 in dilution buffer (10 mM Tris-HCl, pH 7.5, 10% glycerol, 1 mM PMSF, and one protease inhibitor EDTA-free tablet (Roche) pr 50 mL buffer). The diluted samples were loaded onto the equilibrated columns (0.5 mL/min) after which the column was washed with 10 column volumes of wash buffer (10 mM Tris-HCl, pH 7.5, 200 mM NaCl, 20 mM imidazole, 10% glycerol, 1 mM PMSF). IN was eluted using 25 mL elution buffer (10 mM Tris-HCl, pH 7.5, 200 mM NaCl, 150 mM imidazole, 5 mM MgCl_2_, 10% glycerol, 1 mM PMSF). 0.5 mL fractions were collected. After SDS-PAGE analysis, IN containing fractions were pooled and stored in IN storage buffer (200 mM KCl, 10 µM ZnCl_2_, 50% glycerol).

### 2.6. Generation of DNA Acceptor Circles

An approximately 500 bp long PCR product was made using pTrcHis-TOPO as template and primers “Acceptor-circle-fw” and “Acceptor-circle-rv” (see the DNA-oligonucleotides section). The PCR product was cloned into pTrcHis-TOPO using the TOPO^®^ TA Cloning^®^ Kit. The resulting plasmid was cut with PstI and the resulting approximately 500 bp fragment was gel purified (E.Z.N.A.^®^Gel Extraction Kit (Omega Biotek , Norcross, GA, USA)) and circularized using T4 DNA ligase. The covalently closed DNA acceptor circles were purified using illustra GFX PCR DNA and Gel Band Purification Kit.

### 2.7. Rolling Circle Amplification-Based IN Detection

Five fmol of the amine labelled 5′-Amine-Oligo A oligonucleotide (see the DNA oligonucleotide section above) was immobilized to NHS modified glass microscopy slides (CodeLink^®^ Activated slides, Surmodics) as described by the supplier. The HIV LTR was completed by hybridization of the oligonucleotide Oligo B (5 fmol dissolved in a hybridization buffer: 40% formamide, 4× SSC, and 10% glycerol) to the Oligo A-conjugated microscopy slide for 30 min. at 37 °C in a humidity chamber. After hybridization, the slides were washed for 1 min. in wash buffer A (0.1 M Tris-HCl pH 7.5, 150 mM NaCl, and 0.3% SDS) and 1 min in wash buffer B (0.1 M Tris-HCl pH 7.5, 150 mM NaCl, and 0.05% Tween-20). The slides were then dehydrated with 96% Ethanol for 1 min.

To bind IN to the immobilized LTR substrate, the LTR modified slide was incubated with or without 1.5 pmol of purified IN in a reaction buffer containing 20 mM pH 6.2 MES (2-(N-morpholino) ethanesulfonic acid), 200 mM KCl, 10 mM MnCl_2_, 10 mM MgCl_2_, 10 mM DTT, and 10% glycerol. The slide was incubated for 30 min on ice and 15 min at room temperature. The slide was then washed in reaction buffer for 15 min to allow integration of the immobilized LTR into the DNA acceptor circles, 50 fmol of DNA acceptor circles in a buffer containing 20 mM pH 6.2 MES, 200 mM KCl, 10 mM MnCl_2_, 10 mM EDTA, and 10% glycerol were added to the LTR functionalized microscopy slides, and incubated with or without 10 mM DTT for 2 h at 37 °C in a humidity chamber. The slides were washed 1 min in wash buffer A, 1 min in wash buffer B (see above), and 1 min in 96% ethanol.

Rolling circle amplification (RCA) mediated detection of acceptor circles bound to the immobilized oligonucleotides was done using Phi29 polymerase mediated amplification of the DNA circle and subsequent detection of the RCA product using a detection probe (see the DNA-oligonucleotides section) essentially as described previously [1]. The RCA products were visualized using a fluorescence microscope (Olympus IX73). For each experimental condition, nine images were taken and the number of signals per microscopic image frame counted using the ImageJ software as described for the DNA adhesion assay.

## 3. Results and Discussion

### 3.1. DTT Creates Single Stranded Nicks in Covalently Closed DNA Circles

**Figure 1:**
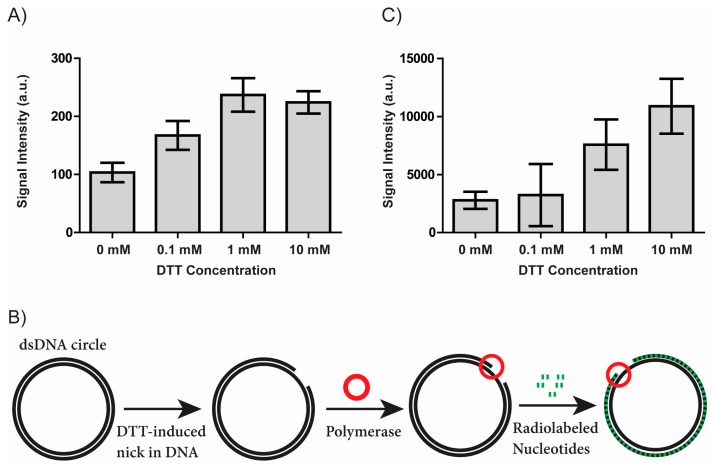
DTT mediated nicking of double stranded DNA. (**A**) Bar chart showing the results of incubating plasmid DNA with varying concentrations of DTT and separating the reaction products in an agarose gel. The chart shows the results of quantifying the bands representing nicked plasmid. Error bars represent the standard error of mean (*n* = 4); (**B**) Schematic depiction of a modified nick translation assay for detection of DNA nicks. DTT mediated nicks in a double stranded plasmid are detected by DNA polymerase (red circle) mediated incorporation of radiolabeled nucleotides (green) initiated at the DNA nicks, if the nicks expose a free 3’- OH end. The polymerase uses the intact DNA circle as a template and the exposed 3’-OH end carrying DNA molecule as a primer; (**C**) Bar chart showing the results of the modified nick translation assay outlined in (**B**). The results are shown as raw values arising from the quantification (arbitrary units). Error bars represent the standard error of mean (*n* = 4).

Preventing loss of DNA integrity due to buffer additives is important for any assay detecting DNA modification events and becomes imperative when detection of a single or very few DNA molecules is needed. To test the effect of DTT on DNA integrity, we used a standard DNA nicking assay for analysis of DNA. In this assay, supercoiled plasmid DNA was incubated with varying DTT concentrations and subsequently analyzed in an agarose gel as described in the materials and methods section. Nicking of supercoiled plasmid DNA results in a mobility shift compared to intact plasmid DNA (Supplementary Materials Figure S1). The bands representing nicked plasmid were quantified and the results shown in Figure 1A. Incubating the plasmid DNA with DTT resulted in a relatively weak, yet detectable, dose dependent increase in the intensity of the band representing nicked plasmid from 103 (arbitrary units) in the absence of DTT to 167, 237, and 224 when the plasmid was incubated with 0.1, 1, or 10 mM DTT respectively (mean of four independent experiments). This result shows that DTT is able to introduce nicks in double stranded DNA. Interestingly, this effect was observed even without any added copper, which was previously thought necessary for thiol mediated nicking of DNA [11,15,16].

To further elucidate the nature of the DTT induced DNA nicks, we set up a second nick-sensing experiment, a modified nick translation assay, which is schematically depicted in Figure 1B. Intact plasmid DNA was incubated with DTT and subsequently incubated with Taq DNA polymerase. In the case of a nick exposing a free 3′-OH end, the Taq DNA polymerase can incorporate radiolabelled nucleotides using the intact circle as template and the 3′-OH carrying DNA molecule as a primer. Subsequently, the DNA was bound to a nylon membrane and the amount of incorporated radiolabelling was visualized using a phosphorimager and quantified using ImageJ.

The results obtained using this modified nick translation assay are shown in Figure 1C. The signal intensity arising from samples incubated without DTT was 2780 (arbitrary units). The signal intensity rose upon incubation with 0.1, 1, and 10 mM DTT to 3228, 7571, and 10878, respectively (mean of four independent experiments). The samples incubated with 0.1 mM DTT did not show a significant increase in signal intensity when compared to samples not treated with DTT. A negative control incubated with 10 mM DTT but not incubated with Taq polymerase was included (data not shown). In this sample, it was not possible to detect any radiolabelling on the membrane demonstrating that the assay is specific for detection of polymerase-mediated incorporation of radiolabelled nucleotides. In addition to confirming the ability of DTT to introduce nicks in DNA without added catalyst, the results of the modified nick translation assay suggests that at least a subset of the DTT generated nicks contain a 3′-OH group.

In the present study, we focus on the consequences of thiol mediated nicking of DNA under typical experimental conditions. For this reason, the presented experiments display two key differences from most of the previous literature on the DNA-cleaving activity of thiols, since these studies have mainly focused on elucidating the mechanisms behind the reaction pathway [9,16,23,24,25]. Firstly, our results were obtained without the deliberate addition of copper and secondly, the thiol concentrations used in this study range from 0.1 mM to 10 mM DTT, whereas the DNA cleavage activity in previous studies was mostly shown with micromolar concentrations of thiols [9,16,23,24,25], along with catalytic copper. Supplementary Materials Table S1 illustrates that DTT concentration in the low mM range reflects the composition of common storage and reaction buffers used for DNA modifying enzymes.

### 3.2. DTT Facilitates the Immobilization of DNA to NHS-Ester Coated Microscopy Slides

**Figure 2:**
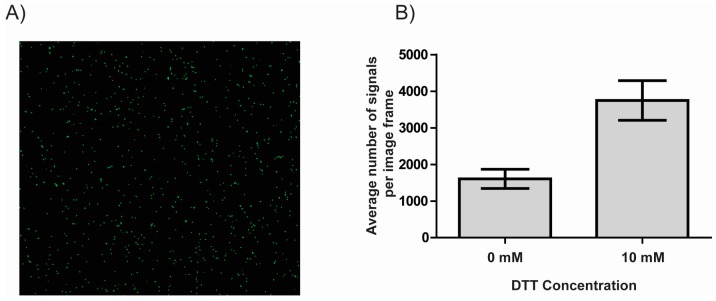
DTT mediated immobilization of double stranded DNA. (**A**) Representative image of immobilized fluorescent DNA molecules visualized using fluorescence microscopy; (**B**) Bar chart showing the results of exposing an NHS-ester modified microscopy slide to fluorescently labelled linear DNA either in the absence of DTT or in the presence of 10 mM DTT. Immobilized DNA molecules were visualized using fluorescence microscopy and quantified using ImageJ. The results shown are the number of DNA molecules visible per image frame. Error bars represent the standard error of mean (*n* = 6).

Immobilization of DNA forms the foundation of many modern DNA sensor studies including fluorescence microscopic, graphene electronic, and plasmon resonance based methods which may be coupled with a polymerase amplification enabled signal amplification step [26,27,28]. A fluorescence microscopy based readout was used to test the effect of DTT on DNA immobilization onto NHS-ester coated glass slides (CodeLink^®^ Activated slides, Surmodics). The functionalized glass slides were incubated with fluorescently labelled 1500 bp PCR product either in the presence or absence of DTT. Using fluorescence microscopy, the amount of DNA immobilized on the microscopy slide could be quantified (Figure 2A). The number of signals per image frame was quantified using ImageJ and the results shown in Figure 2B.

The number of DNA molecules bound to the surface was increased by more than a factor of two from an average of 1606 signals per image frame (no DTT added) to an average of 3748 signals per image frame upon addition of 10 mM DTT (mean of six independent experiments), strongly suggesting that DTT stimulates immobilization of DNA to NHS-ester functionalized slides and certainly may influence results obtained using such surfaces.

### 3.3. DTT Influences the Outcome of a RCA DNA Sensor System

**Figure 3:**
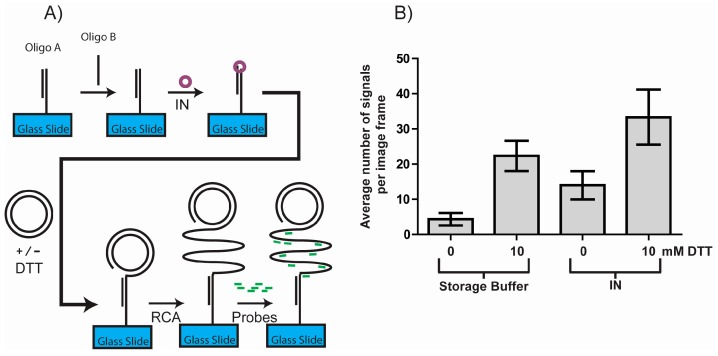
The influence of DTT on the outcome of a DNA sensor system based assay for detection of IN activity. (**A**) Schematic depiction of a novel method for detecting IN (see results and discussion for details); (**B**) The results of the assay outlined in (**A**) when performed either with (the two rightmost columns) or without (the two leftmost columns) IN. The assay was performed with or without addition of 10 mM DTT during the circle immobilization step as indicated on the graph. Individual RCA products were visualized using fluorescence microscopy and their number was quantified using ImageJ. The results shown are the number of DNA molecules visible per image frame. Error bars represent the standard error of mean (*n* = 4).

In order to further investigate the influence of DTT on single molecule detection assays, we used the Rolling Circle amplification Enzyme Activity Detection (REEAD) assay recently developed for ultrasensitive detection of retroviral integrase (IN) activity [22]. A schematic depiction of this assay is shown in Figure 3A. In short the IN REEAD assay relies on the enzyme mediating integration of a surface bound DNA fragment into a closed DNA circle [22]. In theory, only when the target, IN, is present will this reaction occur and generate a 3′-OH DNA end that can facilitate RCA. RCA in turn generates long tandem repeat products that can be visualized at the single molecule level by hybridization of specific fluorescent probes and the results obtained by counting the number of signals using a fluorescence microscope. Interestingly, as evident from Figure 3B, DTT alone had a significant effect comparable to the effect of IN. Both in the presence and the absence of IN, the number of signals per image frame rose with approximately the same number (19 and 18 respectively) upon addition of DTT. DTT thus markedly increased the background, and thereby reduced the signal to noise ratio, creating serious problems for the assay readout.

These results clearly highlight the importance of meticulously ensuring consistent reaction conditions as well as to be aware of the possibility that DTT may cause unintended side reactions altering the outcome of single molecule detecting experiments and other ultrasensitive assays.

## 4. Conclusions

The present study demonstrates important effects of DTT on DNA. Using standard molecular biological reaction conditions, we show that DTT is able to introduce single stranded nicks in double stranded DNA. Furthermore, DTT was able to facilitate immobilization of fluorescently labelled DNA to a functionalized microscopy slide. Although being too modest to substantially affect the results of traditional bulk assay setups, the effect of DTT was strong enough to severely affect the results of a recently developed single molecule detection REEAD setup, demonstrating a potential impact on single molecule detection protocols.

Unlike previous studies investigating thiol mediated DNA effects, the reported results were obtained using reaction conditions without any added copper. It can, however, not be ruled out that the DTT mediated reactions might be catalyzed by trace amounts of Cu^2+^ in the utilized ddH_2_O [29,30] or commercial buffers. Nevertheless, the presented results point to serious problems posed by DTT in various novel single molecule studies of DNA or DNA reactions. Our findings underscore the importance of carefully ensuring uniformity of reaction conditions when performing single molecule studies and of being aware of buffer additives in general and DTT in particular.

## Supplementary Materials

The following are available online at http://www.mdpi.com/1424-8220/17/6/1201/s1, Figure S1: The ability of DTT to introduce nicks in plasmid DNA was investigated in a classical nicking assay. In this assay, we utilized the fact that nicked DNA is separated from supercoiled and relaxed DNA when electrophoresed in the presence of the DNA intercalating dye ethidium bromide. This is due to the fact that ethidium bromide binding introduces positive supercoiling in relaxed plasmid DNA, which results in an increased mobility. Nicked plasmid DNA retains the mobility of relaxed DNA even in the presence of ethidium bromide, since the introduced overwinding escapes via the nick. Figure S1 shows a representative image obtained from gel electrophoretic analysis of plasmid DNA incubated with increasing amounts of DTT (lanes 2–4) or no DTT (lane 1). The high mobility bands represent intact plasmid DNA and the retarded bands represent nicked plasmid. Table S1: Overview of the concentration of DTT in the storage buffers of commonly used DNA modifying enzymes as well as in their reaction buffers. * note the storage buffer of Exonuclease III contains 5 mM of the thiol beta-mercaptoethanol.

## Abbreviations

bp	Base pairs
ddH_2_O	Double distilled water
DTT	Dithiothreitol
EDTA	Ethylenediaminetetraacetic acid
IN	Retroviral integrase
IPTG	Isopropyl β-d-thiogalactoside
NHS	N-Hydroxysuccinimide
PCR	Polymerase Chain Reaction
PMSF	phenylmethylsulfonyl fluoride
RCA	Rolling circle amplification
REEAD	Rolling circle amplification enzyme activity detection
Tris	Tris(hydroxymethyl)aminomethane
