# Supplementary material for: The Effects of Dithiothreitol on DNA
Source: Sensors (Basel). 2017 May 24;17(6):1201. doi: 10.3390/s17061201 (PMC5492665; doi:10.3390/s17061201)
Supplement: Supplementary file 1 [file sensors-17-01201-s001.pdf]

Supplementary Materials: The Effects of Dithiothreitol on DNA

Søren Fjelstrup, Marie Bech Andersen, Jonas Thomsen, Jing Wang, Magnus Stougaard, Finn Skou Pedersen, Yi-Ping Ho, Marianne Smedegaard Hede and Birgitta Ruth Knudsen

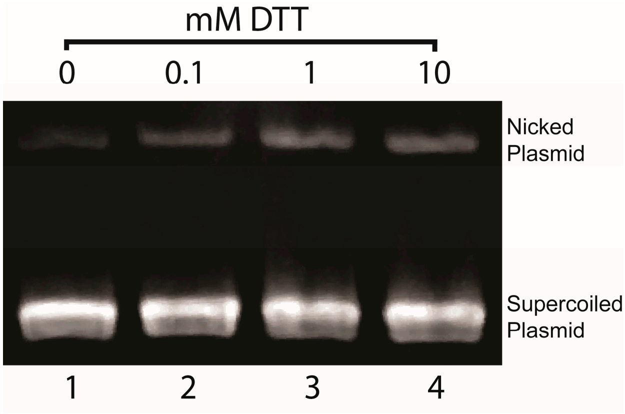

Figure S1. The ability of DTT to introduce nicks in plasmid DNA was investigated in a classical nicking assay. In this assay we utilized the fact that nicked DNA is separated from supercoiled and relaxed DNA when electrophoresed in the presence of the DNA intercalating dye ethidium bromide. This is due to the fact that ethidium bromide binding introduces positive supercoiling in relaxed plasmid DNA, which results in an increased mobility. Nicked plasmid DNA retain the mobility of relaxed DNA even in the presence of ethidium bromide, since the introduced overwinding escapes via the nick.

Figure S1 shows a representative image obtained from gel electrophoretic analysis of plasmid DNA incubated with increasing amounts of DTT (lanes 2–4) or no DTT (lane 1). The high mobility bands represent intact plasmid DNA and the retarded bands represent nicked plasmid.

Table S1. Overview of the concentration of DTT in the storage buffers of commonly used DNA modifying enzymes as well as in their reaction buffers. *note the storage buffer of Exonuclease III contains 5 mM of the thiol beta-mercaptoethanol.

Enzyme	Storage Buffer	Reaction Buffer	Product Number	Company
**Taq Polymerase**	1 mM	0 mM	B9014S	New England Biolabs
**Phi 29 Polymerase**	1 mM	4 mM	M0269S	New England Biolabs
**Exonuclease III**	0*	1 mM	M0206S	New England Biolabs
**NEB Buffer 1,2,3,4**	n/a	1 mM	B700(1-4)S	New England Biolabs
**Calf Intestinal Phosphatase**	0 mM	10 mM	P4978	Sigma Aldrich
**T4 Kinase**	1 mM	5 mM	M0201S	New England Biolabs
**HiFi Taq DNA Ligase**	1 mM	10 mM	M0647S	New England Biolabs
**T4 Ligase**	1 mM	10 mM	M0202S	New England Biolabs
